# Anterior superior iliac spine fracture following DCIA free flap harvest: A two-case series and surgical recommendations

**DOI:** 10.1016/j.jpra.2026.05.013

**Published:** 2026-05-12

**Authors:** Eladio Marcelo Samudio Scavone, Prakash Patel, Rajinikanth Janakiraman

**Affiliations:** Royal Darwin Hospital, 105 Rocklands Dr, Eladio Marcelo Samudio Scavone 45 Grevillea Cct, Darwin, Australia

**Keywords:** DCIA flap, Iliac crest, ASIS fracture, Mandibular reconstruction, Donor-site morbidity

## Abstract

**Summary and Keywords:**

The deep circumflex iliac artery (DCIA) free flap is widely used for mandibular reconstruction due to its favourable bone stock and contour. However, donor-site morbidity remains a concern. Anterior superior iliac spine (ASIS) fracture is a rare but under-recognized complication.

**Cases:**

We present two cases of ASIS fracture following DCIA flap harvest. In both patients, the fracture occurred in the early postoperative period following mobilization and presented with acute hip pain and gait disturbance. Imaging confirmed ASIS fracture. The preserved bone bridge between the ASIS and osteotomy site measured 16.1 mm and 20.6 mm, respectively. One patient required surgical removal of the fractured ASIS while the other patient was managed conservatively with satisfactory functional recovery.

**Discussion:**

ASIS fracture following iliac crest harvest is thought to result from structural weakening of the iliac crest combined with traction forces from the sartorius and tensor fascia lata muscles. Biomechanical studies demonstrate reduced iliac crest strength when the osteotomy is performed within 20–25 mm of the ASIS. Preservation of *a* ≥ 2–3 cm anterior bony bridge is therefore recommended. Although prophylactic donor-site fixation has been described, current evidence is limited. Most non-displaced fractures can be managed conservatively.

**Conclusion:**

ASIS fracture is a rare but preventable complication of DCIA flap harvest. Maintaining an adequate distance from the ASIS and cautious postoperative mobilization are key strategies to reduce risk.

## Introduction

Deep circumflex iliac artery free flap (DCIA) is an excellent option for mandibular and maxillary reconstruction following oncologic resection, benign pathology, or osteoradionecrosis. Either as vascularized free tissue transfers or as a non-vascularized bone graft. Its advantages include favorable bone height, natural curvature, and the potential for dental rehabilitation, making it optimal for head and neck reconstruction.^1^

The three types of iliac bone fracture are classified as follows:

Type A: The fracture runs anteroinferior.

Type B: The fracture line runs posteriorly along the iliac crest.

Type C: Mixed fracture lines type A and type B^2^

Although the literature shows different percentages of complications, including vascular and neurological injuries, herniation of abdominal organs, accidental perforations, and rarely, iliac crest fracture. Ascertaining the real morbidity of anterior iliac crest bone graft harvesting is difficult because different techniques are used for harvesting and complications are measured differently, the reported incidence of iliac crest fracture ranges from 0 to 4.1 %.^3^

Anterosuperior iliac spine fracture is probably caused by the sudden contraction of the outer muscles in a weakened iliac crest structure.^4^ Intraoperative fractures require reduction and fixation, whereas postoperative fractures can usually be conservatively managed with physiotherapy.^5^

The main donor site morbidity in the vascularized iliac crest bone free flap is pain (27 %); however, early complications included cellulitis (3.6 %), deep vein thrombosis (3.6 %), and femoral nerve neuropraxia (4.8 %). Late complications > 6 months include anterolateral thigh sensitivity (27 %), contour irregularity of the basin (20 %), and hernias (9.7 %)^6^

We present two cases of ASIS fracture following DCIA flap harvested and discuss the underlying mechanism and preventive surgical strategies.

## Case presentation

### Case 1

A 33-year-old male was diagnosed with a midline mandible ameloblastoma underwent a midline segmental mandibulectomy with left DCIA free flap reconstruction. Vascular anastomosis was performed to the facial artery and external jugular vein. The patient had an athletic body type (BMI: 24.9). The postoperative course was complicated with surgical seroma collection which was managed conservatively. On postoperative day 10, the patient reported sudden onset left hip pain associated with gait disturbance. Postoperative CT imaging confirmed a type A ASIS fracture along with suspected collection in the neck. He was taken back to the theatre for the surgical site infection in the neck. During the same procedure, the left iliac donor site was explored, revealing a large seroma and a mobile bony fragment consistent with an avulsed ASIS.

### Case 2

A 77-year-old male left mandible osteoradionecrosis, in the background of a previously treated left tonsillar squamous cell carcinoma, underwent left segmental mandibulectomy with reconstruction using a left DCIA free flap. Vascular anastomosis was performed to the facial artery and common facial vein. The patient had a low body weight (BMI: 20.1).

Approximately 1 month postoperatively, the patient developed progressive left hip pain, associated with gait disturbance and weakness during activities as squatting. Clinical examination revealed a palpable, mobile swelling over the left iliac crest.

## Discussion

The deep circumflex iliac artery (DCIA) flap remains a valuable option for mandibular reconstruction due to its favorable bone height, contour, and ability to support dental rehabilitation. However, donor site morbidity remains a recognized limitation, including pain, sensory disturbance, abdominal wall complication, and rarely, iliac crest fracture.^5^

Donor site pain is the most common complication, with an incidence of approximately 3.5 %–27 %.^5^ The damage done to the iliac bone by the osteotome appears to play an important role when the osteotome is applied. Various types of cracks can occur, and the osteotome should be applied at right angles to the iliac bone, with the blade gently hitting the bone.^6^

Anterior superior Iliac spine (ASIS) fracture is an uncommon but clinically significant complication following iliac crest harvest. The underlying mechanism relates to structural weakening of the anterior iliac crest, combined with traction forceps exerted by the sartorius and tensor fascia latae muscles during early ambulation. This creates a stress riser at the weakened bone, predisposed to either avulsion-type or linear crest fractures. Compared with the fibula free flap, the vascularized iliac crest flap demonstrates superiority on higher anatomical adaptation and donor site morbidities, such as ankle rigidity or instability, in defects > 12, the use of iliac crest flaps has not been encouraged because fractures may occur due to stress applied to the lower limb^6^

Sensory changes following harvesting of the iliac crest bone have been reported to be present in up to 38 % of cases, the majority of them being caused by injuring the 1) latero cutaneous branch of the subcostal nerve 2) lateral cutaneous branch of the iliohypogastric nerve, and the lateral femoral cutaneous nerve of the thigh. Attempts should be made to preserve these structures through careful dissection and preservation.^7^

Three options can significantly decrease the risk of iliac crest fracture fatigue following bone graft harvesting from the anterior iliac wing. The iliac crest should be left intact and harvested below the ASIS, which would significantly relieve the stress peak. It also balances the stress distribution along the iliac wing. From the biomechanical point of view, longer bone grafts are preferable over deeper bone grafts, and a harvest site located at least 20–25 mm posterior to the ASIS should be preferred to minimize the risk of iliac fatigue fracture. Possibly, plate fixation over the harvest area will prevent fatigue and iliac wing fracture, although there are not sufficient studies to prove it, most of the authors advocate this pathway.^8^

The downward pull of the attachments of the sartorious muscle and tensor fascia latae on the iliac crest could be the cause of ASIS stress fracture. According to the literature, there are significant differences in crest strength with graft removal at 3 cm or 1.5 cm of the ASIS. Removal of bone 3 cm posterior to the ASIS preserves approximately 2.4 times more strength of the iliac crest than bone removal at 1.5 cm, leaving the sartorius and tensor fascia latae with maximal support.^8^

## Conclusion

ASIS fracture is a rare but important donor-site complication following DCIA flap harvest. Preservation of an adequate anterior iliac crest bone bridge, ideally ≥2–3 cm from the ASIS, appears to be a key technical factor in minimizing fracture risk. Early recognition of postoperative donor-site pain is essential to allow prompt diagnosis and appropriate management. While most fractures can be managed conservatively, prevention through careful surgical techniques remains paramount.

Complications associated with the free flap of the iliac crest bone were found to be acceptable and well tolerated.

The use of osteotome harvest grafts increases the incidence of cortical cracking compared with the use of an oscillating saw.

Regarding the treatment of an iliac crest fracture only when it is an intraoperative finding or there is a large, comminuted fracture along with small bowel protrusion, an open reduction and internal fixation will be necessary.

**Fig. 1:**
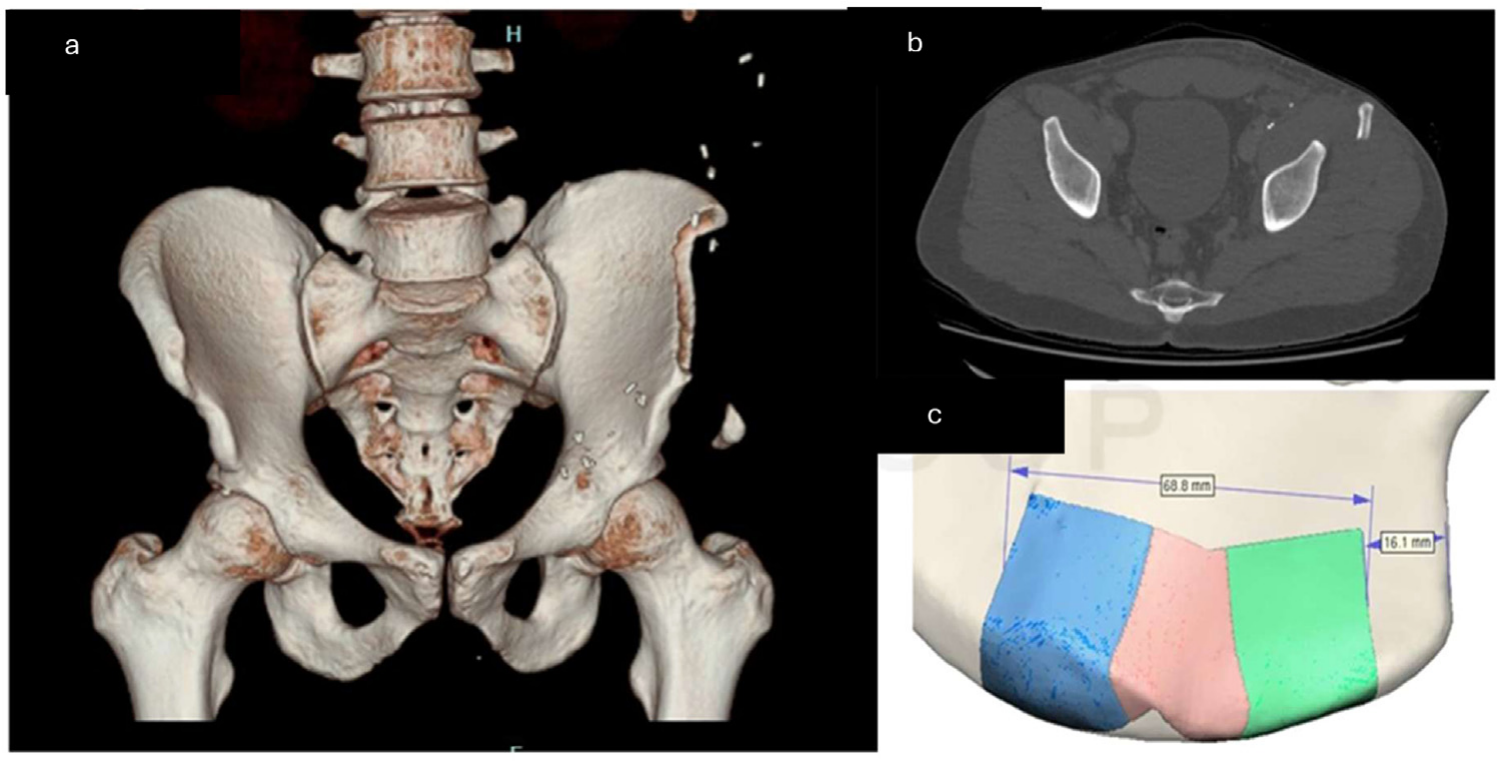
A Postoperative CT scan with bony reconstruction showing type A fracture. Fig. 1B. Avulsion fracture on axial cuts Fig. 1C. Virtual surgical planning demonstrated that 68.8 mm of the iliac bone crest was harvested with only 16.1 mm distance between the ASIS and the anterior osteotomy margin. The fracture was managed conservatively with activity modification and physiotherapy, resulting in satisfactory recovery.

**Fig. 2:**
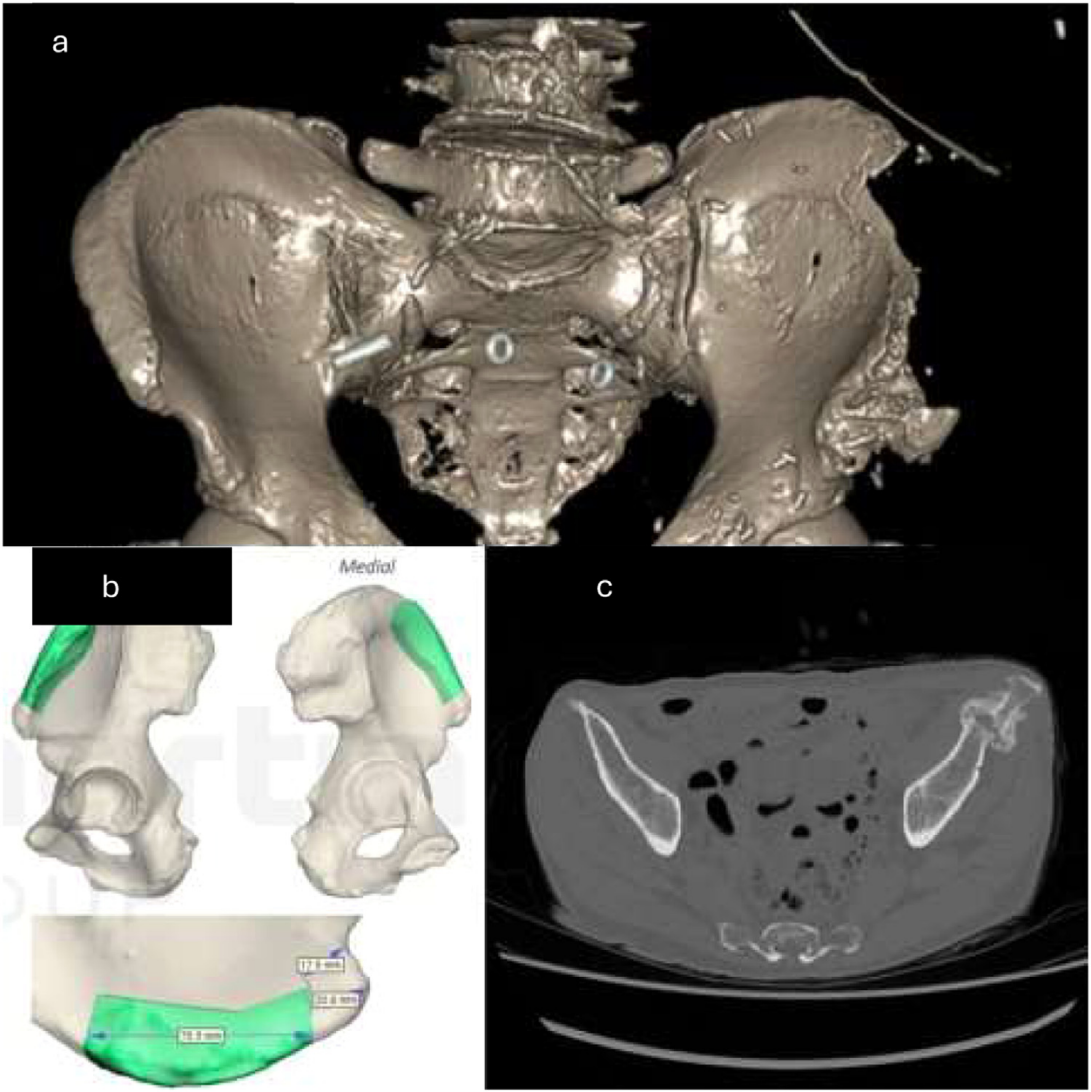
A CT imaging demonstrated an anterosuperior iliac crest fracture (Type A). Fig. 2B. Surgical planning analysis showed that 75.9 mm of iliac crest bone had been harvested, with 20.6 mm distance from the ASIS to the osteotomy site. Fig. 2C. Axial view of the CT scan showing callus formation within the fracture. The patient was managed conservatively following orthopaedic consultation, with physiotherapy and gradual mobilization. He achieved good functional recovery.

**Fig. 3 a:**
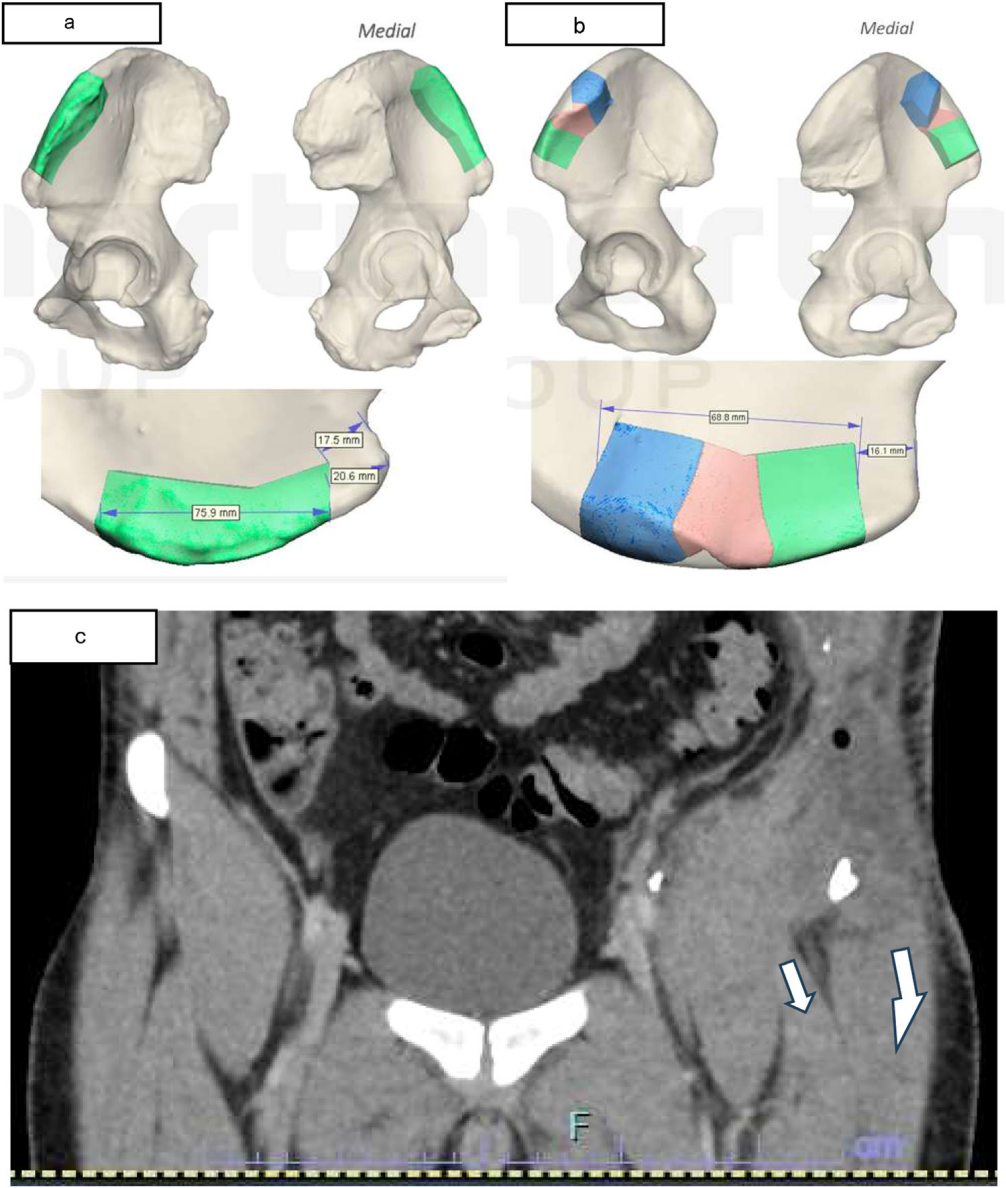
Virtual surgical planning of case 1. b Virtual surgical planning of case 2. c. CT Images showing Sartorius muscle and Tensor fascial latae on white arrows showing a downward traction force.

Conservative management is appropriate in cases where the fracture does not cause significant symptoms (Fig. 1, Fig. 2, Fig. 3 a).

In both cases presented, osteotomy was performed relatively close to the ASIS, with preserved bone bridges of 16.1 mm and 20.6 mm, respectively. These findings are consistent with biomechanical studies demonstrating that harvesting within 20–25 mm of the ASIS significantly reduces iliac crest strength. Furthermore, removal of bone closer to 1.5 cm from the ASIS has been shown to reduce crest strength compared to harvests performed at approximately 3 cm, where significantly greater structural integrity is maintained.

## Funding

Self-Funded.

## Ethical approval

Not required.

## Declaration of competing interest

None.
